# Activation of nuclear factor kappa B (NF-κB) by connective tissue growth factor (CCN2) is involved in sustaining the survival of primary rat hepatic stellate cells

**DOI:** 10.1186/1478-811X-3-14

**Published:** 2005-11-22

**Authors:** Runping Gao, David R Brigstock

**Affiliations:** 1 Center for Cell and Vascular Biology, Children's Research Institute, Columbus Ohio 43205 USA; 2 Department of Surgery, The Ohio State University, Columbus, Ohio 43212 USA; 3 Department of Molecular and Cellular Biochemistry, The Ohio State University, Columbus, Ohio 43212 USA

**Keywords:** connective tissue growth factor, CCN2, hepatic stellate cell, NF-κB, survival, apoptosis, fibrosis

## Abstract

**Background/Aims:**

Connective tissue growth factor (CCN2) is a matricellular protein that plays a role in hepatic stellate cell (HSC)-mediated fibrogenesis. The aim of this study was to investigate the regulation by CCN2 of cell survival pathways in primary HSC.

**Methods:**

Primary HSC were obtained by *in situ *enzymatic perfusion of rat liver. NF-κB activation was assessed by immunoblotting for IκBα phosphorylation and degradation and by NF-κB p50 or p65 nuclear accumulation. NF-κB DNA-binding activity was determined by gel mobility shift assay while NF-κB response gene expression was evaluated using a luciferase reporter. Cell viability was assessed by Trypan blue staining or ATP luminescent assay while apoptosis was evaluated by caspase-3 activity.

**Results:**

CCN2 induced IκBα phosphorylation and degradation as well as nuclear accumulation of NF-κB. Activated NF-κB comprised three dimers, p65/p65, p65/p50 and p50/p50, that individually bound to DNA-binding sites and subsequently triggered transcriptional activity. This was confirmed by showing that CCN2 promoted activity of a NF-κB luciferase reporter. CCN2 promoted survival of serum-starved HSC and protected the cells from death induced by blocking the NF-κB signaling pathway using Bay-11-7082, a specific inhibitor of IκBα phosphorylation.

**Conclusion:**

CCN2 contributes to the survival of primary HSC through the NF-κB pathway.

## Introduction

Hepatic stellate cells (HSC) are the primary targets of fibrogenic stimuli in the injured liver. During the development of fibrosis, HSC undergo a transition from resting vitamin A-rich cells to an activated myofibroblastic phenotype characterized by loss of vitamin A, expression of α-smooth muscle actin, enhanced proliferation and increased production of various extracellular matrix components [1-4]. Activation of HSC has been identified as a central event in hepatic fibrosis and is regulated by a wide variety of molecules including cytokines, cell-surface receptors, signal transduction molecules and factors that regulate HSC gene expression at the transcriptional and post-transcriptional levels [3-6].

Connective tissue growth factor (CCN2, also known as CTGF) is a cysteine-rich matricellular protein that regulates cell adhesion, migration, proliferation, survival, and differentiation [7]. It has fibrogenic properties *in vitro *and is over-expressed in many fibrotic lesions, including those of the skin, lung, kidney and liver [8-12]. CCN2 production is enhanced during progressive activation of primary rat HSC *in vitro *as well as by transforming growth factor-β [11-13]. CCN2 induces migration, proliferation and adhesion of HSC as well as enhanced expression of type I collagen [14-19].

Transcription factor NF-κB is a key regulator of the growth, differentiation, and fate of mammalian cells [20]. NF-κB exists in virtually all cell types and represents a family of inducible transcription factors that are activated by a variety of stimuli including viral infection, lipopolysaccharide, oxidative stress, and cytokines [21]. The active form of NF-κB is found in the nucleus as either a heterodimer or a homodimer composed of five members of the Rel family of proteins (p65, p50, p52, c-Rel, and RelB) [20]. It has recently been reported that transcriptional repressor CBF1 plays a key role in regulating NF-κB activity through its interaction with a dual NF-κB/CBF1-binding site in the IκBα promoter [22,23]. IκBα regulates NF-κB activity by directly interacting with the transcriptional factor to form inactive complexes that are located to the cytoplasm. Following specific signaling, phosphorylation of IκBα at serine 32 and 36 by IκB kinase leads to its ubiquitinylation and degradation by the proteasome, and transport of active NF-κB to nucleus [24]. Active NF-κB is involved in the expression of numerous cytokines, acute phase response proteins, adhesion molecules and Rel/IκB proteins [20]. Also, when NF-κB activation is prevented or inhibited, cells undergo enhanced apoptosis showing that active NF-κB exerts a cytoprotective role by inhibiting apoptosis [25].

Several studies have compared NF-κB activity in quiescent versus activated HSC [26-28]. NF-κB activity is increased in cultured activated HSC but it is not required for either cell proliferation or the process of activation. In contrast, active NF-κB plays an important role in preventing apoptosis of activated HSC [26,29]. Understanding mechanisms of HSC survival may provide the basis for novel anti-fibrotic therapies that focus on the ability to clear activated HSC from the liver by inducing them to undergo apoptosis. Since the principal CCN2 receptor on HSC is integrin α_v_β_3 _[19], which is intimately associated with HSC survival [30], we have investigated the role of CCN2 in NF-κB activation and HSC survival.

## Results

### CCN2 induces phosphorylation of IκBα and translocation of NF-κB

**Figure 1:**
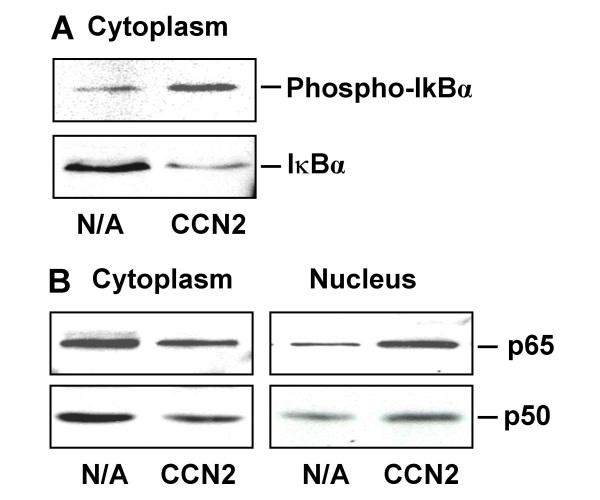
**Effect of CCN2 on stimulation of IκBα phophorylation and NF-κB translocation. **Freshly isolated rat HSC were cultured for 24 h in 5% FBS DMEM and for another 48 h in serum-free medium. The cells were harvested following incubating the cells for 30 min in the absence or presence of 100 ng/ml CCN2, and nuclear extracts were prepared. Western blot analysis shows that CCN2 induces IκB phosphorylation and degradation (A) and translocation of the NF-κB subunits p65/p50 from cytoplasm to nucleus (B).

In most cell types, NF-κB is found in the cytoplasm as an inactive dimer bound to one of the IκB inhibitory proteins (IκBα, IκBβ, or IκBγ) that mask its nuclear localization signal. As assessed by Western blotting of cytoplasmic protein extracts day 4 primary HSC, phospho-IκBα was elevated while total IκBα was decreased following stimulation by CCN2 (Figure 1A), indicating that CCN2 could induce IκBα phosphorylation and degradation. Additionally, following CCN2 stimulation, levels of p65 and p50 were reduced in the cytoplasm but increased in the nucleus (Figure 1B), consistent with the notion that CCN2-induced IκBα phosphorylation and degradation was associated with translocation of cytoplasmic NF-κB to the nucleus.

### CCN2 promotes NF-κB DNA binding activity

**Figure 2:**
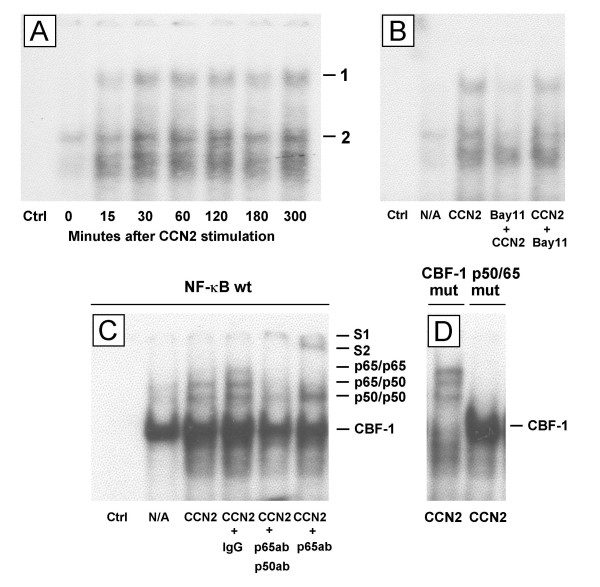
**Modulation of NF-κB DNA binding activity by CCN2**. HSC were harvested at the desired time points after treatment with or without 100 ng/ml CCN2. 6 μg nuclear protein extract were used in 20 μl reactions, containing 0.2 ng ^32^P-labeled double strand NF-κB oligonucleotides. Reactions were fractioned through a nondenaturing 4% polyacrylamide gel. (A) Complex 1 (p65/p50) and complex 2 were enhanced after stimulation with CCN2. "Ctrl" represents a reaction lacking nuclear extract. (B) Bay11-7082 inhibited complex formation when added prior to CCN2 treatment ("Bay 11 + CCN2") but not when added subsequent to a 1 hour pretreatment with CCN2 ("CCN2 + Bay11"). (C) A supershift assay was performed by incubating pre-assembled gel shift assay complexes containing 8 μg nuclear extract with either 2 μg normal rabbit IgG or 2 μg anti-NF-κB antibody prior to separation through 8% polyacrylamide gel, showing that CCN2 stimulates the formation of an anti-p65/p65/anti-p50/p50/NF-κB oligonucleotide (S1) and an anti-p65/p65/NF-κB oligonucleotide (S2) supershift complexes. (D) A gel shift assay was performed following pre-incubating the nuclear extracts with either ^32^P-labeled p50/p65 site mutant oligonucleotides or CBF-1 site mutant oligonucleotides.

To further explore whether active NF-κB can bind to its target DNA sequence and activate gene transcription in response to CCN2 stimulation, NF-κB DNA binding activity was determined by EMSA following incubation of HSC nuclear protein extracts with ^32^P-labeled NF-κB oligomers containing NF-κB/CBF1 binding sites. Two complexes were significantly enhanced by CCN2, reaching a plateau about 30 min after CCN2 addition (Fig. 2A). The NF-κB inhibitor, Bay11-7082, prevented complex formation when added to the cells prior to CCN2 treatment but not when added after CCN2 treatment (Figure 2B). As shown in Figure 2C, active NF-κB induced by CCN2 comprised three separate dimers (p65/p65, p65/p50 and p50/p50) based on the fact that a supershift (S1) was obtained with anti-p65 and anti-p50 antibodies with the concomitant disappearance of all three bands and that a supershift (S2) with anti-p65 antibody was associated with loss of the top two bands. Three dimers of NF-κB induced by CCN2 were also demonstrated following incubation of the nuclear extract with a CBF-1 mutant oligonucleotide probe but not with a p50/p65 mutant oligonucleotide probe (Figure 2D). These results indicate that CCN2 induces activation of NF-κB and its assembly into three dimers that individually bind to NF-κB DNA binding site.

### Effect of CCN2 on expression of NF-κB target genes

**Figure 3:**
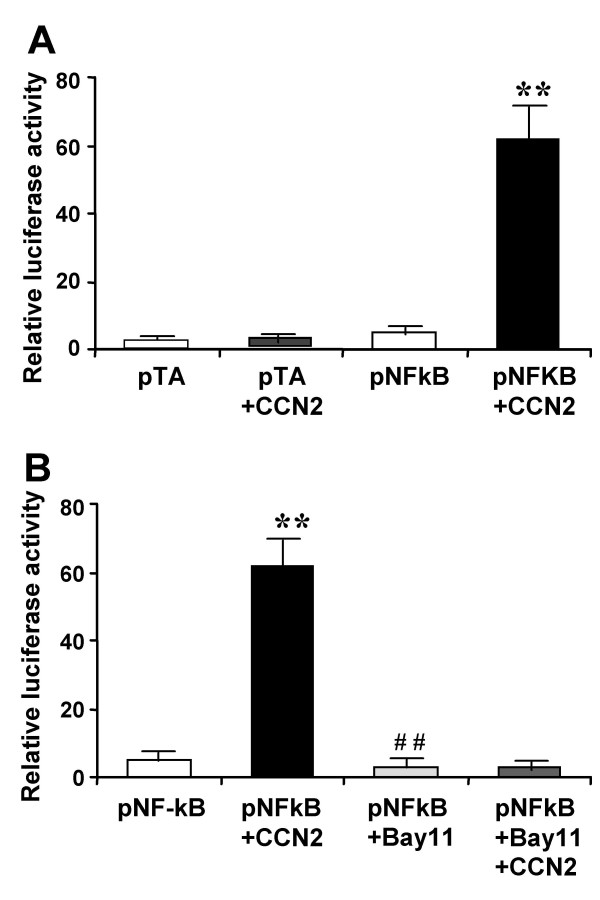
**Effect of CCN2 on expression of NF-κB response genes**. (A) Freshly isolated rat HSC were placed in 12-well plates, transfected with 1 μg pTA-Luc or pNF-κB-Luc, and then incubated for another 24 h in the absence or presence of 100 ng/ml CCN2. (B) Transfected cells were pre-treated with 10 μM Bay11-7082 for 30 min and cultured for another 24 h in the absence or presence of 100 ng/ml CCN2. **P < 0.01 vs. pNF-κB-Luc; ^##^P < 0.01 vs. pNF-κB-Luc + CCN2.

To explore the effect of CCN2 on transcriptional signaling by NF-κB, day 4 primary HSC cultures were transfected with luciferase reporter genes driven by either a minimal promoter alone (pTA) or together with four tandem copies of the NF-κB DNA binding sites (pNF-κB) that were identical to those used for the EMSA studies. pNF-κB generated 1.5-fold higher luciferase activities than pTA in HSC, whereas, the luciferase activities of pNF-κB in the cells following stimulation with CCN2 were 13-fold higher than control cells (Figure 3A). CCN2-mediated elevation of pNF-κB luciferase activity was completely abrogated by treatment of the cells with Bay11-7082, a specific inhibitor of IκBα phosphorylation (Figure 3B), suggesting that CCN2 modulates the transcriptional and translational event of NF-κB target genes via NF-κB signaling pathway.

### CCN2 sustains HSC survival through NF-κB signaling pathway

**Figure 4:**
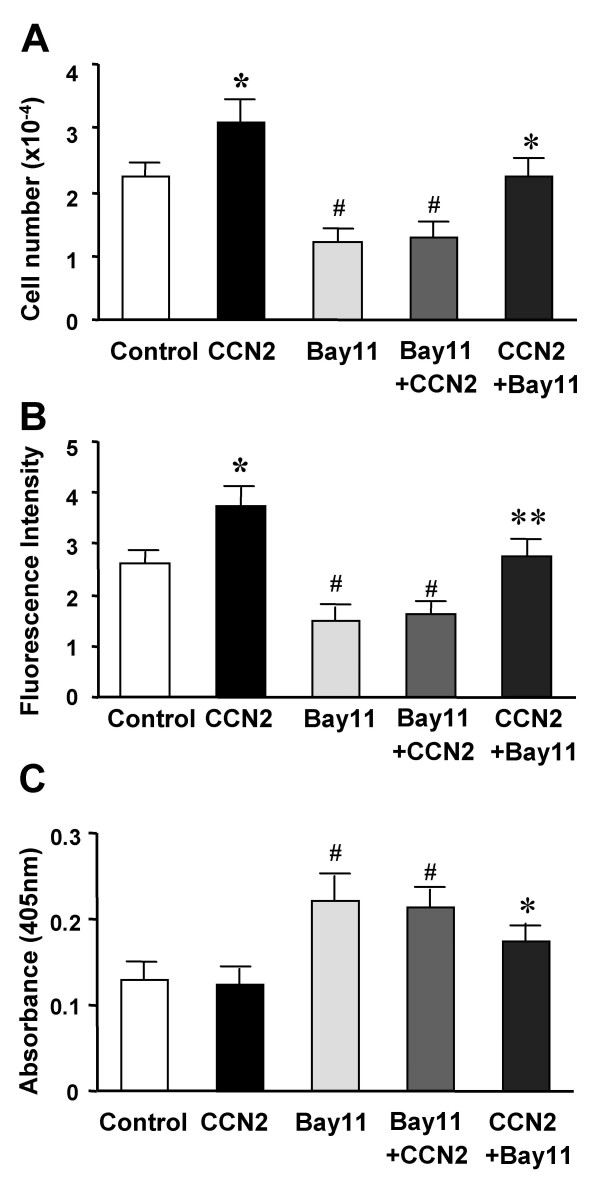
**Effect of CCN2 in sustaining HSC survival. **Freshly isolated rat HSC were cultured in 6-well plates in 5% FBS DMEM for 24 h, followed by serum deprivation for 48 h. The cells were cultured for another 24 h in the absence or presence of 100 ng/ml CCN2 ("CCN2"). In the CCN2 protection assay, the cells were incubated with 10 μM Bay11-7082 for 24 h alone ("Bay11") or following pre-treatment of the cells with CCN2 for 1 h ("CCN2+Bay11"). For the Bay11 blocking assay, the cells were pre-treated with Bay11-7082 for 30 min and cultured for another 24 h in the presence of 100 ng/ml CCN2 ("Bay11+CCN2"). At the end of incubation time period, (A) cells were trypsinized and survival was determined by Trypan blue exclusion, or (B) cell viability was also quantified by measurement of the fluorescence intensity using CellTiter-Glo™ reagent, or (C) cell apoptosis was assessed by measurement of caspase-3 activity at 405 nm using a luminescence assay kit. *P < 0.05 vs. control; ^#^P < 0.05 vs. CCN2 group; **P < 0.01 and * P < 0.05 vs. Bay11-7082 group.

To examine the effect of CCN2 on the fate of HSC, day 4-primary HSC cultures were treated with or without CCN2 for 24 h. Cell viability was determined by Trypan blue exclusion or by luminescent assessment of cellular ATP levels. As shown in Figures 4A and 4B, each assay showed that HSC viability was significantly elevated by CCN2. To determine if the NF-κB pathway was involved in this effect, Bay11-7082 was added to HSC cultures. As mentioned above, when added prior to CCN2, the inhibitor completely blocked the ability of CCN2 to stimulate NF-κB DNA binding activity whereas it had little inhibitory effect on DNA binding activity in cells that has been pre-treated with CCN2 (Figure 2B). While Bay11-7082 inhibited cell survival as expected, it had little inhibitory effect in cells that had been pre-treated with CCN2 (Figure 4A,B), consistent with the data shown in Figure 2B and supportive of the notion that prior stimulation of NF-κB by CCN2 was sufficient to overcome the effects of subsequently blocking NF-κB with Bay 11. Similarly, Bay-11-induced caspase-3 activity in HSC was reduced as much as 25% in CCN2-stimulated cells, consistent with the ability of CCN2 to rescue cells from apoptosis (Figure 4C).

Collectively, these data suggest that CCN2 is a survival factor for HSC and that survival is regulated via NF-κB signaling.

## Discussion

CCN2 has emerged as a key mediator of fibrosis in both acute and chronic diseases [8-12]. In the liver, CCN2 expression is associated with hepatic fibrosis in both human subjects and animal models [10-12,14,17,31]. CCN2 appears to be directly involved in HSC biology as it is produced as a function of activation or exposure the cells to various fibrogenic stimuli including transforming growth factor-β, platelet-derived growth factor, alcohol and acetaldehyde [14]. Additionally, CCN2 promotes HSC adhesion, migration, proliferation, and synthesis of collagen type I [14,19], all of which are properties of activated HSC. [1-4,32]. Activation of HSC is regulated by several soluble factors, including growth factors, cytokines, and products of oxidative stress, as well as by extensive changes in the composition and organization of the extracellular matrix. HSC activation has previously been linked to activation of NF-κB while over-expression of IκBα in HSC has been shown to suppress NF-κB activation [26-29]. Our data show that serum-starved day 4 HSC demonstrate very low levels of nuclear NF-κB and no detectable DNA-binding activity. Following CCN2 stimulation, a marked nuclear translocation of NF-κB was evident along with a persistent DNA binding activity of its three dimers, and an induction of IκBα phosphorylation and degradation. Collectively, these data show that CCN2 activates the NF-κB signaling pathway in HSC. Moreover, as assessed using a NF-κB reporter construct, we showed that NF-κB response gene expression is induced by CCN2 in day 4 cultured HSC and support previous findings that NF-κB response genes are induced in activated HSC [26,29].

Apoptosis has been described as the nexus between liver injury and fibrosis [33] and increasing evidence suggests that NF-κB is involved in survival pathways in multiple hepatic cell types. For example, NF-κB prevents hepatocyte apoptosis following liver regeneration or exposure to tumor necrosis factor-α [34-36]. In addition, NF-κB also protects hepatocarcinoma cells or activated HSC from apoptosis [26,29,37]. Consistent with this latter observation, we showed that CCN2 promoted HSC survival and that NF-κB was involved in the response as shown by the finding that pretreatment of HSC with CCN2 protected the cells from Bay11-7082-induced decreased cell survival and increased caspase-3 activity.

The ability of CCN2 to sustain HSC survival supports previous observations showing that CCN2 is a survival factor for other cell types such as endothelial cells or chicken embryo fibroblasts [38,39]. Additionally, the related molecule, CCN1 (also known as CYR61) promotes anti-apoptotic pathways when over-expressed in breast cancer MCF7 cells in an integrin-dependent manner [40], consistent with the recognition of integrins as signaling receptors for CCN proteins [41]. In the case of HSC, the principal CCN2 receptor is integrin α_v_β_3 _[19] which transduces survival signals in activated HSC [30] as well as during angiogenesis, wound healing, osteoporosis, and tumor metastasis [42-51]. Apoptosis in HSC is inhibited by engagement of integrin α_v_β_3 _[30] and disruption of integrin-mediated HSC adhesion leads to induction of apoptosis [52]. Since expression of CCN2 and integrins is enhanced during HSC activation and liver fibrosis [53-57], persistence of the activated fibrogenic phenotype in HSC may occur, at least in part, by NF-κB survival pathways that are triggered via the binding of CCN2 to its integrin α_v_β_3 _receptor.

Activation of HSC is also associated with the expression of death receptors such as Fas and TRAIL-R2, suggesting that HSC fate is likely determined by a balance between survival and apoptotic stimuli [33]. For example, HSC undergo apoptosis following treatment with nerve growth factor, a response that is due to the expression of the p75 nerve growth factor receptor [58] which has recently been implicated as a CCN2 signaling molecule in kidney mesangial cells [59]. Furthermore, pro-apoptotic effects of CCN2 have been reported in vascular smooth muscle cells and breast cancer cells, although the underlying mechanisms have yet to be understood [60-62]. Thus, depending on the presence and activity of its cognate cell surface receptors and their associated signaling pathways, CCN2 may be able to drive either apoptosis or survival in HSC. This points to a complex scenario whereby CCN2 may exert apparently opposing or contradictory effects on HSC viability, and future investigations will need to clarify this issue. Nonetheless, clinical fibrosis is now regarded as a largely reversible process that is strongly linked to apoptosis of activated HSC [33,63] and our data showing that CCN2 can promote HSC survival via NF-κB provide support for the development of new anti-fibrotic strategies that target CCN2, its receptors, or its signaling pathways.

## Conclusion

In addition to promoting HSC fibrogenesis [17], CCN2 confers a survival advantage on HSC which is attributable, at least in part, to its ability to activate NF-κB signaling pathways in the cells.

## Methods

### Isolation and culture of HSC

In a protocol approved by the Institutional Animal Care and Use Committee of Children's Research Institute, Columbus, OH, primary HSC were isolated from normal male Sprague-Dawley rats as described [19]. Cells were grown in Dulbecco's modified Eagle's medium (DMEM; Gibco, Grand Island, NY, USA) supplemented with 10% fetal bovine serum (FBS), 100 U/ml penicillin and 100 μg/ml streptomycin. Cells were placed in 20 × 100 mm cell culture dishes (Falcon; Becton Dickinson, Franklin Lakes, NJ, USA) for nuclear extraction, 6-well tissue culture plates (Falcon) for Western blot and for cell viability assays, 12-well tissue culture plates (Falcon) for luciferase reporter gene transfection. The cells were then cultured DMEM/5% FBS for 24 h, followed by serum-free medium for 48 h. On day 4, the cells were treated with or without 100 ng/ml CCN2, and harvested at the desired time points. Human recombinant 38 kDa CCN2 was produced in a Chinese hamster ovary cell expression system as described [15].

### Electrophoretic mobility shift assay (EMSA)

Nuclear extracts were prepared as described [64]. ^32^P-end-labeled double-stranded oligonucleotide probes used in this study comprised either wild type NF-κB oligonucleotide (sense: 5'-tgaggggactttcccagg-3'), p50/p65 mutant oligonucleotide (sense: 5'-tgaggcgactttcccagg-3') or CBF1-mutant oligonucleotide (sense: 5'-tgaggggacttcccgagg-3') [23]. The double-stranded NF-κB oligmers were used in nuclear protein-DNA binding reactions (20 μl volume) in which 1 μg poly dI:dC and 6 μg nuclear protein extract were incubated for 20 min at 4°C prior to addition of 0.2 ng ^32^P-labled double-stranded oligonucleotide for 30 min at 4°C. The contents of each tube were electrophoresed on non-denaturing 4% polyacrylamide gels which were then dried and analyzed by autoradiography. Supershift assays were performed by incubating pre-assembled gel shift assay complexes containing 8 μg nuclear extract with either 2 μg rabbit normal IgG, 2 μg rabbit polyclonal anti- p65 NF-κB IgG or/and 2 μg rabbit polyclonal anti- p50 NF-κB IgG (Santa Cruz Biotechnology Inc, CA, USA) for 2 h at 4°C before electrophoresis. The samples were then electrophoresed on 8 % polyacrylamide gels [65].

### Transfections and luciferase assay

HSC were transfected with pNF-κB-Luc or pTA-Luc control vector using Superfect transfection reagent (QIAGEN, Valencia, CA, USA) under serum-free conditions for 3 h. The transfected cells were incubated for another 24 h in the absence or presence of 100 ng/ml CCN2. After normalization of transfection efficiency by β-galactosidase expression, luciferase enzyme activity was then quantified using a reporter assay kit (Clontech, Palo Alto, CA, USA).

### SDS-PAGE and immunoblotting

25 μg cytoplasmic or nuclear extracts (see above) were subjected to SDS-PAGE in 5–15% gradient gels at 120 V for 1.5 h. Proteins were transferred to nitrocellulose membranes which were individually incubated with 1:500 dilutions of rabbit anti-IκBα, -phospho-IκBα, -NF-κB p65, or -NF-κB p50 polyclonal IgG (Santa Cruz Biotechnology Inc, CA, USA) in 5% nonfat milk TBST for 24 h at 4°C. The filters were then incubated with 1:1000 dilutions of HRP-conjugated goat anti-rabbit IgG for 1 h at room temperature. The membrane was washed extensively before detection using chemiluminescence.

### Cell viability assay

Freshly isolated HSC were placed in 6-well tissue culture plates and incubated in DMEM/5% FBS for 24 h followed by serum-free medium for 48 h. The cells were incubated for an additional 24 h in the absence or presence of 100 ng/ml CCN2. In CCN2 protection assays, 10 μM Bay11-7082 was added to the medium either alone or following treatment of the cells with CCN2 for 1 h. At the end of the incubation period, the cells were trypsinized, mixed 1:1 with Trypan Blue solution (Sigma) and counted within 3 minutes under light microscopy using a hemocytometer.

In an alternative approach for measurement of cell viability, the CellTiter-Glo™ Luminescent assay kit was employed to assess the relative levels of cellular ATP. At the end of the incubation period, cells were treated with CellTiter-Glo™ reagent according to the manufacturer's instructions. Fluorescence was measured using black/clear tissue culture plates (BD Biosciences, Bedford, MA, USA). Cell viability was quantified by measurement of the sample fluorescence intensity at 560_EX_/590_EM_.

### Caspase-3 activity assay

Freshly isolated HSC were placed in 12-well tissue culture plate, and incubated in DMEM/5% FBS for 24 h followed by serum-free medium for 48 h. The cells were incubated for an additional 24 h in the presence or absence of 100 ng/ml CCN2. 10 μM Bay11-7082 was added to the medium either alone or following treatment the cells with CCN2 for 1 h. Protein extracts were prepared following manufacturer's instructions. Caspase-3 activity was measured using an assay kit (Promega, Madison, WI, USA) in which cell extracts were mixed with Ac-DEVD-pNA substrate for 1-hour incubation at 30°C in 96-well microtiter plates prior to colorimetric measurement of p-nitroanilide product at 405 nm.

### Statistical analysis

Data are presented as mean ± SE. Differences were analyzed statistically with paired sample student's t-test.

## Competing interests

The author(s) declare that they have no competing interests.

## Authors' contributions

RG carried out the experiments and wrote the draft manuscript. DRB participated in the experimental design and edited the manuscript.
